# The roles of FHL2 as a mechanotransducer for cellular functions in the mechanical environment

**DOI:** 10.3389/fcell.2024.1431690

**Published:** 2024-07-26

**Authors:** Yukari Fujimoto, Naotaka Nakazawa

**Affiliations:** ^1^ Graduate School of Science and Engineering, Kindai University, Higashiosaka, Japan; ^2^ Department of Energy and Materials, Faculty of Science and Engineering, Kindai University, Higashiosaka, Japan

**Keywords:** FHL2, substrate rigidity, LIM domain protein, gene expression, mechanotransduction

## Abstract

The cell has multiple mechanisms for sensing and responding to dynamic changes in the mechanical environment. In the process, intracellular signaling is activated to modulate gene expression. Recent studies have shown that multifunctional signaling molecules that link intracellular force and gene expression are important for understanding cellular functions in the mechanical environment. This review discusses recent studies on one of the mechanotransducers, Four-and-a-half LIM domains 2 (FHL2), which localizes to focal adhesions (FAs), actin cytoskeleton, and nucleus. FHL2 localizes to FAs and the actin cytoskeleton in the cell on stiff substrate. In this situation, intracellular tension of F-actin by Myosin II is critical for FHL2 localization to FAs and actin stress fibers. In the case, a conserved phenylalanine in each LIM domain is responsible for its localization to F-actin. On the other hand, lower tension of F-actin in the cell on a soft substrate causes FHL2 to be released into the cytoplasm, resulting in its localization in the nucleus. At the molecular level, phosphorylation of specific tyrosine in FHL2 by FAK, non-receptor tyrosine kinase, is critical to nuclear localization. Finally, by binding to transcription factors, FHL2 modulates gene expression for cell proliferation as a transcriptional co-factor. Thus, FHL2 is involved in mechano-sensing and -transduction in the cell in a mechanical environment.

## 1 Introduction

Mechanical environments, such as substrate rigidity, substrate topography, and spatial confinement, are signals for the intracellular pathways in cell growth and differentiation (Vogel and Sheetz, 2006; Vining and Mooney, 2017; Sheetz, 2019; Chaudhuri et al., 2020). Intracellular signaling pathways activate the expression of downstream genes, which modulate cellular responses. Thus, intracellular signaling activated by the mechanical environment is critical to understanding how the mechanical environment leads to gene expression through mechanosensing mechanisms.

Four-and-a-half-LIM domain 2 (FHL2) is a LIM domain (domain discovered in the proteins, Lin11, Isl-1, and Mec-3) family protein that contains a specific structure with a tandem zinc-finger motif (Kadrmas and Beckerle, 2004; Anderson et al., 2021). Although a general zinc-finger motif binds directly to DNA, the LIM domain mediates protein-protein interaction. Previous studies have suggested that FHL2 mediates intracellular signaling under the control of cytoskeletal regulators (Müller et al., 2002; Schiller et al., 2011). Importantly, FHL2 shuttles between the cytoplasm and the nucleus in this process. Previous studies have reported that a transcriptional co-factor, such as YAP/TAZ and MRTF/MAL, works as a mechanotransducer by shuttling between the cytoplasm and the nucleus (Miralles et al., 2003; Dupont et al., 2011; Er et al., 2022). FHL2 is the first molecule reported as an adhesion protein that translocates from the adhesion site to the nucleus in response to changes in extracellular matrix stiffness and intracellular contractility (Nakazawa et al., 2016). Regarding the relationship between FHL2 and cancer phenotypes, the expression level of the *FHL2* gene is upregulated in metastatic cell lines (Kleiber et al., 2007; Zhang et al., 2023). In addition, previous studies have reported that ectopic expression of *FHL2* gene correlates with a pathological phenotype of tumor progression and growth in human patients, mice, and rats (Ding et al., 2009; Hua et al., 2016; Jin et al., 2016; Cai et al., 2018). This suggests that understanding the function of FHL2 may be potentially important for cancer therapy. Taken together, the function of FHL2 as a mechanotransducer appears to be important for understanding the links between mechanical effects on adhesion complexes and gene expression in the cancer cell, which may contribute to new insights for cancer therapy. Here, we discuss recent work on FHL2 functions as a mechanotransducer for cell proliferation as it relates to cancer cell dynamics in a mechanical environment.

## 2 FHL2 mediates mechanical signaling from adhesion sites to the nucleus, leading to gene expression

FHL2 at FAs binds directly to focal adhesion components, including several integrins and FA kinase (FAK) (Wixler et al., 2000; Gabriel et al., 2004; Samson et al., 2004). This suggests that FHL2 is a potential scaffolding protein at FAs. A previous study indicates that the cell without FHL2 shows impairment of FA maturation (Park et al., 2008). However, this effect is due to the lack of ECM proteins through the modulation of gene expression (Park et al., 2008). Since knocking out of the FHL2 gene affects the expression of downstream genes, it is still unclear whether FHL2 itself contributes to adhesion assembly.

To distinguish the functions of FHL2 as a scaffolding component and a transcriptional co-factor, it might be necessary to elucidate the function of each FHL2 domain on FA and to perform rescue experiments with each domain in FHL2 in a knockout condition. Another way to identify the functions of FHL2 in FA may be to combine perturbation for FHL2 function and optogenetic tools such as chromophore-assisted light inactivation (CALI) (Liao et al., 1994; Ryu et al., 2021; Takemoto, 2021). Thus, it remains unclear how FHL2 contributes to FA maturation, but recruitment of FHL2 to FAs is Myosin II activity-dependent (Kuo et al., 2011; Wolfenson et al., 2016). Since conserved mechanisms of the LIM domains facilitate its localization to the actin filament, FHL2 recruitment may occur at the end of FA maturation (Sun et al., 2020; Winkelman et al., 2020). Although FHL2 silencing reduces cancer cell migration and invasion, there is no direct evidence that FHL2 contributes to cell migration and invasion as an adhesion component (Brun et al., 2013; Hua et al., 2016; Wang et al., 2020; Jiao et al., 2022). However, distinguishing the function of FHL2 may be important when considering FHL2-targeted cancer therapy, as a strategy of cancer therapies generally depends on the stage of cancer, in which case FHL2 may have different functions at different cancer stages (Umar et al., 2012; Klein, 2020).

**FIGURE 1:**
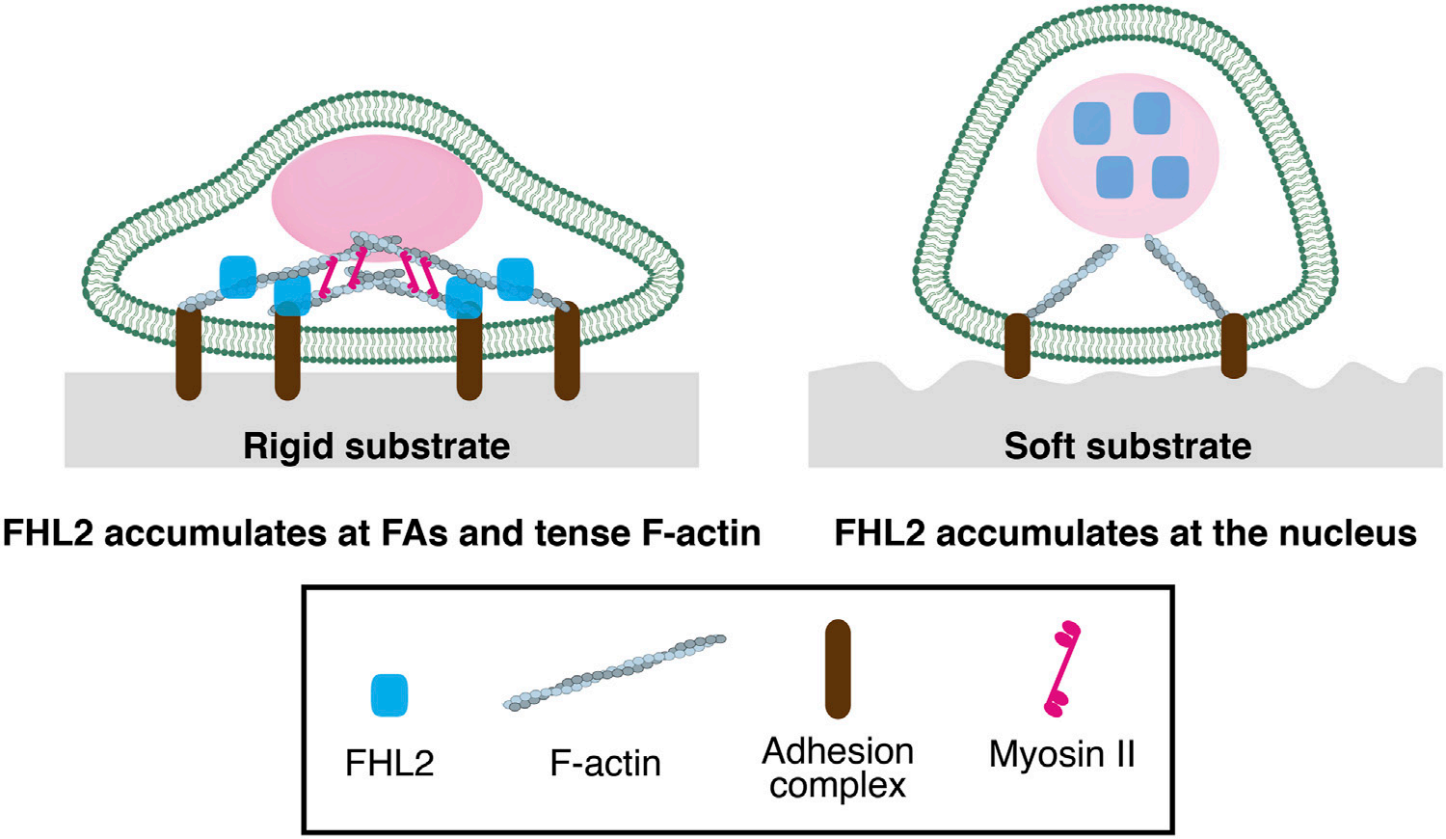
The functions of FHL2 as a mechanotransducer in the cell on a rigid or soft substrate (Left) FHL2 localizes at Focal adhesion or tense F-actin in the cell on rigid substrate (Right) FHL2 localizes to the nucleus on soft substrate, resulting in its binding to a specific promoter of the gene with transcriptional factor for cell proliferation.

As mentioned above, FHL2 localization at FAs is dependent on Myosin II activity (Kuo et al., 2011). This suggests that intracellular tension of F-actin is critical for FHL2 localization to FAs. A rigid substrate generally facilitates FA maturation through tense actin stress fibers. Our previous study demonstrated that FHL2 accumulates at FAs in the cell on the rigid substrate (Figure 1) (Nakazawa et al., 2016). Conversely, soft substrate facilitates the release of FHL2 into the cytoplasm, resulting in its localization to the nucleus (Figure 1). Since cells with perturbation of Myosin II activity of actin polymerization show nuclear localization of FHL2, cell contraction is critical for FHL2 localization. Our study found that cyclic stretch can rescue FHL2 nuclear localization by the soft substrate (Cui et al., 2015; Nakazawa et al., 2016). In this case, intracellular tension is important for FHL2 localization in the nucleus rather than the disassembly of FA.

FA kinase (FAK) is a non-receptor tyrosine kinase that plays critical role in the maturation of FA (Dawson et al., 2021; Le Coq et al., 2022). Previous studies have confirmed the biochemical interaction between FAK and FHL2 (Gabriel et al., 2004; Nakazawa et al., 2016). FAK phosphorylates Y93 in FHL2, which facilitates the nuclear localization of FHL2 dependent on substrate rigidity or intracellular tension. Other tyrosines, with the exception of Y93, are phosphorylated by another non-receptor tyrosine kinase, c-Abl. Therefore, phosphorylation of Y93 in FHL2 by FAK appears to be specifically dependent on intracellular force and substrate rigidity (Wang G.-F. et al., 2021).

Following its shuttling to the nucleus, FHL2 co-localizes with the active RNA polymerase (Pol) II on the promoter sequence of *p21*/*CDKN1A* gene. Since *p21*/*CDKN1A* is a negative regulator of cell proliferation, thus FHL2 modulates cell proliferation through *p21*/*CDKN1A* gene expression in a force- or substrate-rigidity-dependent manner (Nakazawa et al., 2016). However, the molecular mechanisms of FHL2 nuclear transport remain unclear so far. Since FHL2 is a small protein (32.2 kDa), active transport to the nucleus might not be required. One possibility is that phosphorylation of FHL2 by FAK might enhance its concentration at the active RNA pol II site. Recent studies on phase separation of the molecules suggest that protein modification, including phosphorylation, impacts transcriptional machinery (Hnisz et al., 2017; Boija et al., 2018; Sabari et al., 2018). It is notable that LIMD1, another LIM domain protein, has been reported to contribute to FA matulation through phase separation (Wang Y. et al., 2021). Further research is required to elucidate the effect of chemical modification in FHL2 on its accumulation at the specific region in the nucleus.

## 3 FHL2 binds to F-actin directly in a tension-dependent manner

Previous studies have reported that FHL2 localizes to F-actin. A recent study observed that some LIM domain family proteins, including FHL2, HIC5, and Zyxin, are recruited to tense actin fibers (Sun et al., 2020). Surprisingly, direct binding to tense actin fibers depends on a conserved phenylalanine among these LIM domain proteins (Sun et al., 2020; Sun and Alushin, 2023). Furthermore, FHL2 with mutations at specific amino acid residues (F80A, F141A, F200A, F263A) accumulates at the nucleus in the cell on a rigid substrate (Sun et al., 2020). These findings indicate that FHL2 functions as a mechanosensor of actin fibers, not only as a mechanotransducer.

## 4 Discussion and conclusion

In this review, we summarized the functions of FHL2 as a mechanotransducer and a mechanosensor in the cell in a mechanical environment. FHL2 localizes to FA in the cell on a rigid substrate dependent on intracellular tension through F-actin. At the same time, FHL2 recruitment is facilitated to tense F-actin. FHL2 is released from FAs and F-actin when intracellular tension becomes low on a soft substrate, which leads to shuttling to the nucleus. Thus, FHL2 works as a transcriptional co-factor in the nucleus to modulate gene expression for cell proliferation in a tension-dependent manner.

To understand how FHL2 mediates mechanical signaling, the overall picture of proteins interacting with FHL2 still needs to be identified (Tran et al., 2016). Since binding partners of FHL2 may be different at several locations in the cell on soft/rigid substrates, proteome analysis focusing on the binding partners of FHL2 in the cell on different substrate rigidity might be important to understand how FHL2 functions as a multifunctional protein in a force-dependent manner. In this sense, a technique to check a history of protein-protein association, such as BioID, TurboID, and AirID, might be a useful method to identify the binding partner of FHL2 in different situations (Choi-Rhee et al., 2004; Roux et al., 2012; Branon et al., 2018; Kido et al., 2020). Previous studies using BioID focusing on components of FA have provided a new insight into the interaction between scaffolding proteins (Dong et al., 2016; Chastney et al., 2020; He et al., 2023). However, screening of proteins that interact with FHL2 in different mechanical environments has yet to be performed.

As mentioned, FHL2 in the nucleus modulates *p21*/*CDKN1A* gene expression for cell proliferation in a force- or substrate rigidity-dependent manner (Nakazawa et al., 2016). Although some FHL2 target genes are reported, other downstream genes that are regulated by the mechanical environment in FHL2 dependent manner have not been explored (Morlon and Sassone-Corsi, 2003; Martin et al., 2007; Labalette et al., 2008; Wong et al., 2012; Ng et al., 2014; Dahan et al., 2017). Comparison of transcriptome analysis with and without FHL2 in different mechanical environments will offer further understanding of FHL2 functions for cancer metastasis from a mechanobiological viewpoint.
